# The Interleukin-1α Precursor is Biologically Active and is Likely a Key Alarmin in the IL-1 Family of Cytokines

**DOI:** 10.3389/fimmu.2013.00391

**Published:** 2013-11-20

**Authors:** Busun Kim, Youngmin Lee, Eunsom Kim, Areum Kwak, Soyoon Ryoo, Seung Hyeon Bae, Tania Azam, Soohyun Kim, Charles A. Dinarello

**Affiliations:** ^1^Department of Medicine, University of Colorado Denver, Aurora, CO, USA; ^2^Department of Medicine, Pusan Paik Hospital, College of Medicine, Inje University, Busan, South Korea; ^3^Laboratory of Cytokine Immunology, Department of Biomedical Science and Technology, Konkuk University, Seoul, South Korea; ^4^Department of Internal Medicine, Division of Rheumatology, College of Medicine, University of Ulsan, Seoul, South Korea

**Keywords:** IL-1 family cytokine, precursor IL-1α, DAMPs, sterile inflammation, recombinant protein

## Abstract

Among the 11 members of the IL-1 family cytokines, the precursors of IL-1α, IL-1β, and IL-33 have relatively long N-terminal pro-sequences of approximately 100 amino acid residues prior to the N-terminus of the mature forms. Compared to the mature forms secreted from the cell, 80–90% of the primary translation product is in the intracellular compartment in the precursor form. However, the precursors are readily released from cells during infections but also with non-infectious conditions such a hypoxia and trauma. In this setting, the precursors act rapidly as “alarmins” in the absence of a processing mechanism to remove the pro-sequence and generate a mature form. In the case of IL-1α, the release of the precursor activates adjacent cells via receptor-mediated signaling. However, there are no data comparing the specific activity of the IL-1α precursor to the mature form. In the present study, we compared the precursor and mature forms of recombinant human IL-1α, IL-1β, and IL-33 proteins on the induction of cytokines from A549 cells as well as from human peripheral blood mononuclear cells (PBMC). Similar to the mature form, the IL-1α precursor was active in inducing IL-6 and TNFα, whereas the precursor forms of IL-1β and IL-33 were not active. On PBMC, precursor and mature IL-1α at 0.04 and 0.2 nM were equally active in inducing IL-6. Given the fact that during necrotic cell death, the IL-1α precursor is released intact and triggers IL-1 receptors on tissue macrophages, these data identify the precursor form of IL-1α as a key player in sterile inflammation.

## Introduction

Among the 11 members of IL-1 family cytokines, 10 have no signal peptide, the exception being the IL-1 receptor antagonist (IL-1Ra) (1). The signal peptide in IL-1Ra allows the cell to release the antagonist as an active molecule without a requirement for processing of the IL-1Ra precursor whereas all other members of the IL-1 family require processing from an inactive precursor to an active cytokine. Of these 10 leaderless forms, IL-1α, IL-1β, and IL-33 each have pro-pieces of approximately 100 amino acid residues at their respective N-termini, whereas the other members of IL-1 family cytokines, have shorter pro-pieces, the IL-36 subfamily has the shortest. IL-1α was the first cytokine shown to translocate to the nucleus and the pro-piece contains a nuclear localization sequence (LKKRRL), which binds to chromatin and acts as transcription factor (2–4). Although during apoptosis, the intracellular IL-1α precursor binds tightly to chromatin (4), during necrosis, the precursor dissociates from the chromatin is released from the cell in the precursor form (4). The IL-33 precursor also acts as a transcription factor and is chromatin bound (5); IL-33 is also released from dying cells where extracellular processing takes place by serine proteases (6, 7). The precursor forms for IL-1β and IL-18 are inactive; processing to an active cytokine can take place intracellularly by caspase-1 but extracellularly by neutrophil-derived proteinase-3 (8–10).

Damage (or danger)-associated molecular pattern molecules (DAMPs) are nuclear or cytosolic proteins from necrotic cells or tissues, dying tumor cells or released from cells by an infectious process. DAMP’s released from the cells can activate surface receptors on distant or adjacent cells. Because DAMP’s rapidly initiate inflammation cell in the absence of proper secretion mechanisms, the term “alarmin” is often used for categorizing such endogenous molecule as proposed by Oppenheim (11). The released intracellular proteins have immediate effects on cells expressing receptors involved in inflammation, host defense, and tissue repair. Pathogen-associated molecular patterns (PAMPs) initiate inflammation by an infectious pathogen, most commonly via Toll-Like Receptors (TLR) (12).

In many ways, the hallmark of DAMP activity is sterile inflammation with neutrophil recruitment into the area of the damaged tissues, for example, in myocardial infarction, acute lung injury and cerebrovascular accident (stroke). In acute lung injury, the type II epithelial cells contain constitutive IL-1α precursor and when released, mediates local inflammation, which is prevented by IL-1 receptor blockade (13, 14). In a mouse model of DAMP activity from necrotic tumor cells injected intraperitoneally, there is neutrophilic inflammation but also elevation of hepatic enzyme alanine aminotransferase (ALT) (15). The inflammation was observed in wild-type (WT) mice but not in mice deficient in the IL-1 receptor type 1 (IL-1R1) (15). Furthermore, antibodies to IL-1α rather than to IL-1β blocked the response and the inflammation was still observed in mice deficient in TLR (15). In those studies, the molecular nature of the IL-1α, i.e., whether the precursor or the mature form, was not determined. However, in another study (4) keratinocytes exposed to prolonged hypoxia *in vitro* released cellular contents containing IL-1α with a molecular weight of 30,000 Da consistent with the precursor form. Moreover, *in vivo* neutrophilic response to these unfractionated supernatants was blocked by an IL-1α specific neutralizing antibody (16), suggesting that the IL-1α precursor is active. Since IL-1, either IL-1α or IL-1β, promotes IL-17 production by T cells, alarmin-mediated immune responses may be an important endogenous activator of pathogenic T cells in autoimmune diseases (17, 18).

Because the precursor forms of IL-1α, IL-1β, and IL-33 are released as DAMPs and because the biological responses to DAMP-mediated sterile inflammation plays a major role in many ischemic diseases, we compared the specific biological activities in molar ratio of precursor and mature forms using cytokine responses from a mesenchymal cell (lung type II epithelial cell) and a hematopoietic cell (blood monocytes).

## Materials and Methods

### Recombinant cytokines and antibodies

**Figure 1:**
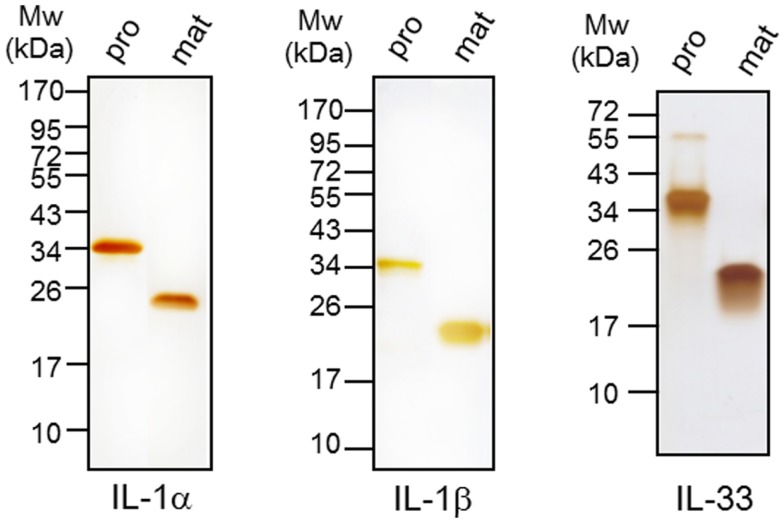
**Expression and purification of recombinant cytokines**. Six different recombinant cytokine proteins were expressed in *E. coli* as indicated. Precursor (pro) IL-1α and mature (mat) IL-1α (200 ng/lane) in left panel, proIL-1β and mature IL-1β (100 ng/lane) in middle panel, and proIL-33 and mature IL-33 (500 ng/lane) were purified by affinity chromatography and then subjected to 10% SDS-PAGE. The purity of each recombinant protein was visualized by silver staining. The data represents one of three independent experiments.

The recombinant proteins were expressed in *E. coli* with six N-terminal histidine tags and purified over a mini-Talon chromatography, ion-exchange for IL-1β or HPLC for IL-1α and IL-33 as described (6). Purity was assessed by silver staining of PAGE (Figure 1). The endotoxin content was less that 1 EU/mg. The N-termini for mature IL-1β was at 117 (caspase-1 site), for mature IL-1α was 115 and for IL-33 111 (19). Anti-human IL-1α was a kind gift of XBiotech (Austin, TX, USA) and IL-1Ra (anakinra) was purchased from R&D Systems, Minneapolis, MN, USA.

### Cell cultures

Human peripheral blood mononuclear cells (PBMCs) were isolated by density centrifugation of blood over Ficoll-Paque™ PLUS (GE, Piscataway, NJ, USA). PBMCs were washed with saline and resuspended in tissue culture medium (RPMI 1640) containing 10% FCS at 5 million cells/mL. The cells were seeded in 100 μL volumes in 96-well flat-bottom microtiter plates and the recombinant cytokines were added in RPMI-10%FCS. In some studies, the recombinant cytokines were preincubated with anti-IL-1α antibody (XBiotech, Austin, TX, USA) before being added to the cells. In other studies, IL-1Ra was added to the cells before the recombinant cytokines. After 24 h of incubation, the supernatants were removed as assayed for IL-6, IL-8, or TNFα using specific ELISA (R&D Systems).

Human A549 cells were cultured as described previously (20). Recombinant cytokines were added in presence or absence of anti-IL-1α or IL-1Ra as described above. After 24 h of stimulation, the levels of IL-6 were measured (R&D Systems). The human mast cell responses to IL-33 were measured in the HMC-1 cell line as described previously (6, 19). After 24 h of incubation with mature or precursor IL-33, IL-8 was measured in the supernatants (R&D Systems).

### Western blots

For detection of precursor and mature IL-1α, 30 μL each of concentrated (×10) overnight A549 cell culture supernatants were used for 10% SDS-PAGE. The samples were transferred to nitrocellulose membrane and the membranes were blocked in 3% BSA/TBST. Anti-IL-1α antibodies (R&D Systems) were used for the western blot. A peroxidase-conjugated secondary antibody (Jackson Immuno Research Laboratories, West Grove, PA, USA) was used to develop the blots along with Supex (Neuronex, Seoul Korea) and an LAS-4000 imaging device (Fujifilm, Japan).

### RT-PCR of IL-1 receptors

Total RNA was isolated with Tri-Reagent (Sigma, St. Louis, MO, USA) from human lung carcinoma A549 cells or PBMCs. A pair of human IL-1R1 sense primer, 5′-GGCTGATAAATGCAAGGAACG-3′ and reverse primer, 5′-CCTAGCACTGGGTCATCTTC-3′, of human IL-1R3 primer, 5′-GAACATCAGCAGGCCCTAGA-3′ and reverse primer, 5′-GGGGAATTGAAACAGCTGTCT-3′, and glyceraldehyde 3-phosphate dehydrogenase (GAPDH) sense primer, 5′-ACCACAGT CCATGCCATCAC-3′ and reverse primer, 5′-TCCACCACCCTGT TGCTGTA-3′, was used for the RT-PCR. MMLV-RT (Beams Bio, Korea) was used for converting 2 μg of total RNA to first strand cDNA, and then PCR reaction was performed at 94°C for 45 s, 70°C for 2 min, and 59°C for 1 min for 30 cycles.

### Statistical analysis

The data is expressed as mean ± SEM. Statistical significance of differences was analyzed by unpaired, two-tailed Student’s *t* test.

## Results

### Precursor IL-1α is active whereas precursor IL-1β is inactive

**Figure 2:**
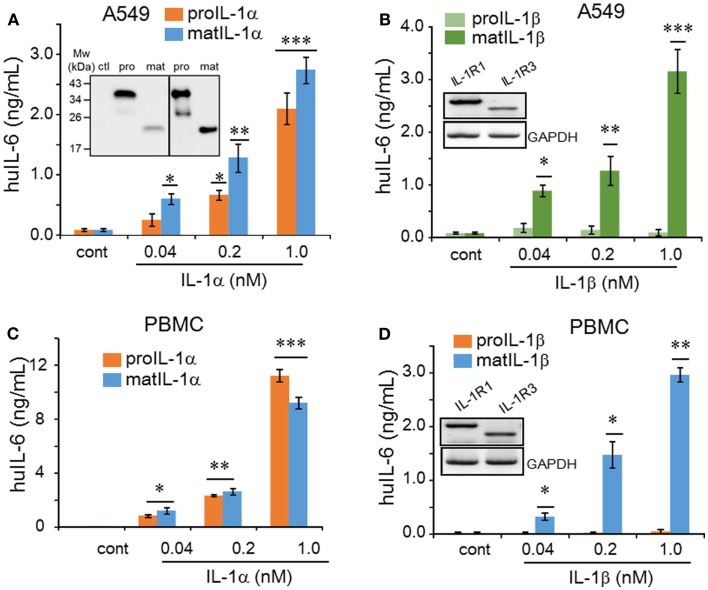
**Comparison of precursor and mature forms of IL-1α and IL-1β on IL-6 production**. **(A)** Mean ± SEM IL-6 supernatant levels induced in A549 cells after 24 h of incubated with IL-1α precursor or mature forms and **(B)** by IL-1β precursor or mature forms at concentrations indicated under the horizontal axis. Inset in **(B)** shows the RT-PCR product of IL-1R1 and IL-1R3 in A549 cells. **(C)** Mean ± SEM IL-6 supernatant levels from PBMCs after 24 h of incubation with IL-1α forms and **(D)** by IL-1β at concentrations indicated under the horizontal axis. Inset in **(D)** shows PCR product of IL-1R1 and IL-1R3 in PBMCs. *p < 0.05; **p < 0.01; ***p < 0.001 compared to control (cont). RT-PCR products shown are representative of one of two independent experiments. IL-6 data from A549 cells shown are one of two independent experiments performed in triplicate. PBMC data are representative of one of two donors performed in triplicate.

We expressed six different recombinant IL-1 family cytokines (precursor IL-1α, mature IL-1α, precursor IL-1β, mature IL-1β, precursor IL-33, and mature IL-33) and purified the recombinant proteins by two-steps of chromatography as described previously (6). Each recombinant cytokine was examined for purity by 10% SDS-PAGE and silver staining, and all six cytokines appeared as primarily a single band (Figure 1). We examined the activity of precursor and mature forms of IL-1α compared to the precursor and mature forms of IL-1β. A549 human lung carcinoma cells were stimulated for 24 h with increasing concentrations of each form. As shown in Figure 2A, precursor and mature forms of IL-1α were active in a dose-dependent manner. Both recombinant proteins induced IL-6, although mature IL-1α was twice as active compared to the precursor at 0.04 or 0.2 nM concentration. The levels reached statistical significance for mature and precursor IL-1α-induced IL-6 at 0.04 or 0.2 nM, respectively. Similar experiments were performed comparing precursor and mature forms of IL-1β. Mature IL-1β was highly active, but precursor IL-1β failed to induce IL-6 production in A549 cells (Figure 2B).

Next we used western blots of the A549 cell supernatant of untreated control, precursor IL-1α, and mature IL-1α in order to demonstrate that the precursor IL-1α-mediated IL-6 production was not due to maturation of precursor IL-1α during overnight treatment. As shown in Figure 2A, precursor IL-1α remained intact (left panel including untreated control, ctl) whereas mature IL-1α decreased when compared to that of recombinant proteins (right panel).

We next compared the biological activity of precursor and mature forms of IL-1α on primary human PBMCs. As shown in Figure 2C, both precursor and mature IL-1α were active and these data are similar to the findings depicted in Figure 2A. However, compared to the activity of mature IL-1α on PBMCs-induced IL-6 production, the induction of IL-6 by mature IL-1β on PBMC was less for each concentration (Figures 2C,D). For example, at 0.04 nM of mature IL-1α-mediated IL-6 from PBMC was 1.5 ng/mL, but that of IL-1β was only 0.2 ng/mL from PBMC. The reduced activity by mature IL-1β on PBMCs is consistent with the presence of the IL-1 receptor-2 on monocytes. The IL-1R2 is non-signaling decoy receptor, which dampens IL-1β driven inflammation (21) and preferentially binds mature IL-1β but not IL-1α (22). In fact, comparing the activity on PBMC and A549 cells, precursor and mature IL-1α induced considerably more IL-6 at all concentrations on PBMC (Figures 2A,C). In the absence of the IL-1R2, IL-1α, and IL-1β signal via the complex of the IL-1R1 and IL-1R3, also known at the IL-1R accessory protein (also known as IL-1R3). Both receptor molecules are expressed in A549 cells and PBMCs, although IL-1R1 expression was more prominent than that of IL-1R3 (Figures 2B,D).

### Precursor IL-1α is neutralized by an anti-IL-1α antibody

**Figure 3:**
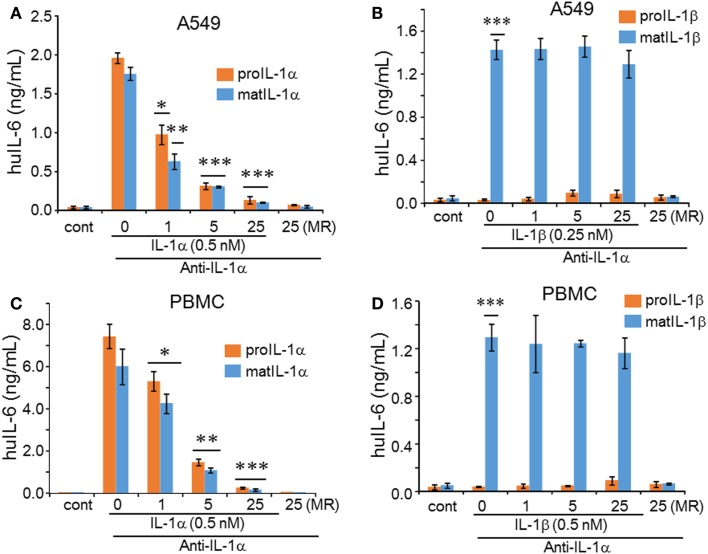
**Inhibition of IL-1 induced IL-6 by precursor and mature forms by anti-IL-1α antibody**. Anti-IL-1α antibody was first incubated with either precursor or mature form of IL-1α or IL-1β before being added to cells. The molar ratio (MR) of antibody to ligand is indicated under the horizontal axis. **(A)** Mean ± SEM IL-6 supernatant levels induced in A549 cells after 24 h of incubated with IL-1α forms and **(B)** by IL-1β forms. **(C)** Mean ± SEM IL-6 supernatant levels from PBMCs after 24 h of incubation with IL-1α forms and **(D)** with IL-1β forms. *p < 0.05; **p < 0.01; ***p < 0.001 compared to control (cont). IL-6 data from A549 cells shown are one of two independent experiments performed in triplicate. PBMC data are representative of one of two donors performed in triplicate.

Considering that the N-terminal domain of the IL-1α precursor contains 115 amino acids, which are not present in the mature form, tertiary folding of the IL-1α precursor may be different from that of the mature form and hence the binding epitopes by anti-IL-1α may not be the same. Therefore, we tested whether the human monoclonal antibody to IL-1α neutralized the precursor form of IL-1α. This antibody is presently be studied in several clinical trials, including type-2 diabetes, psoriasis, and cancer cachexia, as reviewed in Ref. (23). Precursor or mature IL-1α at 0.5 nM was preincubated for 30 min with increasing molar excesses of anti-IL-1α (XBiotech, Austin, TX, USA). The mixtures were then added to A549 cells and after 24 h, the levels of IL-6 were measured in the supernatants. Both precursor and mature IL-1α-induced IL-6 production was inhibited by the antibody (Figure 3A). At equimolar concentrations of antibody to IL-1α, the reduction was approximately 50% for precursor and 60% for mature form. Upon increasing the molar excess of the antibody to fivefold, there was a 80% reduction in IL-1α-induced IL-6 and at a increase to 25, IL-6 decreased further. As expected, mature IL-1β was not inhibited by anti-IL-1α (Figure 3B). Similar to A549 cells assay, precursor and mature IL-1α-induced IL-6 production from PBMC was specifically inhibited by the anti-IL-1α antibody (Figure 3C), whereas mature IL-1β-induced IL-6 from PBMC was not affected by anti-IL-1α (Figure 3D).

### Regulation of TNFα by IL-1α and IL-1β

**Figure 4:**
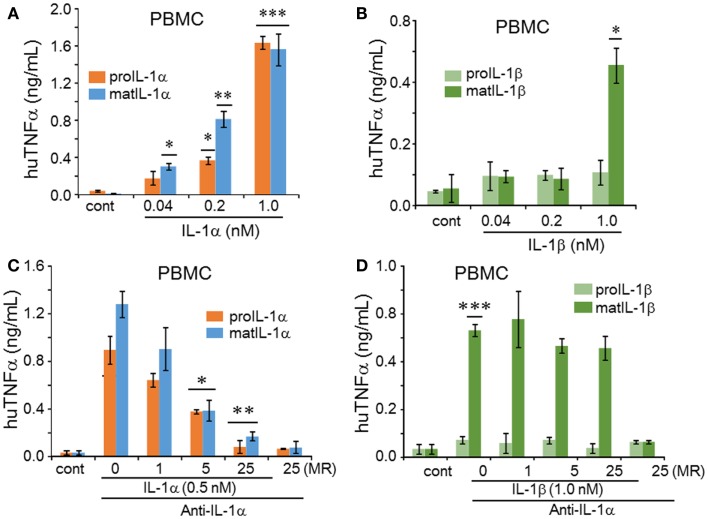
**IL-1α and IL-1β-mediated regulation of human TNFα in PBMCs**. **(A)** The cell culture supernatants of PBMCs treated with precursor or mature recombinant IL-1α from Figure 2C and **(B)** precursor or mature recombinant IL-1β from Figure 2D were used for TNFα levels. Concentrations of the recombinant cytokines are indicated under the horizontal axis. **(C)** The inhibition of anti-IL-1α in precursor or mature recombinant IL-1α-induced TNFα levels **(D)** that of precursor or mature recombinant IL-1β-induced TNFα levels were measured in supernatants from Figures 3C,D, respectively. Data are mean ± SEM; *p < 0.05; **p < 0.01; ***p < 0.001 compared to control (cont). PBMC data are representative of one of two donors performed in triplicate.

Since IL-1 induces TNFα *in vivo* and *in vitro* (24), we next examined the induction of TNFα by precursor and mature forms of IL-1α and IL-1β on PBMC. As shown in Figure 4A, both mature and precursor forms of IL-1α induced TNFα, although the mature form at low concentrations (0.04 and 0.2 nM) induced significantly more TNFα compared to the precursor. Only mature IL-1β induced TNFα from PBMC but this induction was low and only observed at a concentration of 1 nM (Figure 4B). The low induction by mature IL-1β is likely due to the presence of the IL-1R2 on monocytes in the PBMC cultures as mentioned above. Unlike PBMCs, A549 cells failed to produce TNFα when stimulated by either IL-1α or IL-1β at all concentrations (data not shown).

Anti-IL-1α reduced the induction of TNFα by either mature or precursor IL-1α (Figure 4C). In contrast to the effect of this antibody on IL-1α on A549 cells, a molar excess of 5 and 25 was required to neutralize IL-1α activity on PBMC. As shown in Figure 4D, the antibody to IL-1α has no effect on the activity of mature IL-1β.

### IL-1Ra abolishes IL-1α and IL-1β activity

**Figure 5:**
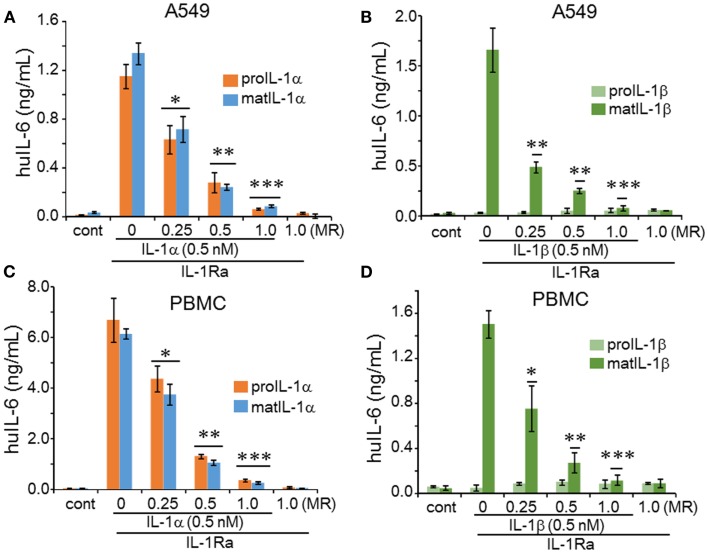
**IL-1Ra inhibition of precursor and mature forms of IL-1α and IL-1β activity**. Prior to stimulation with IL-1 (0.5 nM), increasing concentrations of IL-1Ra were added to cells and are indicated as molar excess of IL-1Ra to IL-1 under the horizontal axis as molar ratio (MR). A549 cells were stimulated with 0.5 nM of precursor or mature recombinant IL-1α **(A)** and 0.5 nM of precursor or mature recombinant IL-1β **(B)** in the presence of absence of IL-1Ra. Similar experiments of human PBMCs were performed with 0.5 nM of precursor or mature recombinant IL-1α **(C)** and 0.5 nM of precursor or mature recombinant IL-1β **(D)**. IL-6 levels in the supernatants represent mean ± SEM; *p < 0.05; **p < 0.01; ***p < 0.001) compared to control (cont). IL-6 data from A549 cells shown are one of two independent experiments performed in triplicate. PBMC data are representative of one of two donors performed in triplicate.

IL-1 receptor antagonist (anakinra) is used to treat a broad spectrum of acute and chronic inflammatory diseases, as reviewed in Ref. (23) and since IL-1Ra blocks the IL-1R1, it remains unknown how many diseases that are controlled by anakinra are due to prevention of IL-1α or IL-1β activity; furthermore, how many diseases are due to the activity of the IL-1α precursor? This is particularly an important issue since little is known of the binding of the IL-1α precursor to the IL-R1 compared to the binding of mature IL-1α, IL-1Ra, and mature IL-1β (25). The rationale for testing the ability of the IL-1Ra to block the responses provides indirect evidence that precursor IL-1α, mature IL-1α, and mature IL-1β share the same receptor since IL-1Ra binds solely to IL-1R1. When we added IL-1Ra to A549 cells at 1:1 molar ratio, IL-6 induction was completely abolished (Figure 5).

**Figure 6:**
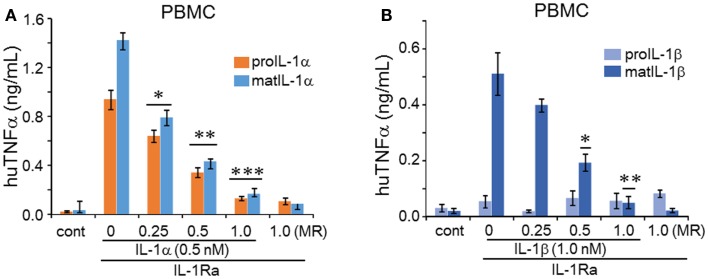
**Inhibition of IL-1α or IL-1β induced TNFα by IL-1Ra**. Prior to stimulation with IL-1α or IL-1β, increasing concentrations of IL-1Ra were added to cells indicated as molar excess of IL-1Ra to IL-1 under the horizontal axis as molar ratio (MR). PBMC cells were stimulated with 0.5 nM of precursor or mature recombinant IL-1α **(A)** and in **(B)** 1.0 nM of precursor or mature recombinant IL-1β. TNFα levels in the supernatants represent mean ± SEM; *p < 0.05; **p < 0.01; ***p < 0.001) compared to control (cont). PBMC data are representative of one of two donors performed in triplicate.

We next reduced the concentrations of IL-1Ra in order to establish a complete dose-response of inhibition by the antagonist. As shown in Figure 5, at a low molar ratio of 0.25, IL-1Ra reduced IL-6 production when stimulated by either the IL-1α precursor, mature IL-1α, or mature IL-1β induced IL-6 production in A549 (*p* < 0.05). The reduction by IL-1Ra of IL-6 production by mature IL-1β in A549 cells (Figure 5B) was more effective than by IL-1α (Figure 5A). Similar experiments with human PBMCs exhibited that IL-1α-induced IL-6 (Figure 5C) and IL-1β-induced IL-6 (Figure 5D) were suppressed by IL-1Ra. In addition, we examined the effect of IL-1Ra on precursor or mature IL-1α and mature IL-1β-induced TNFα production. As shown in Figures 6A,B, TNFα was also inhibited by IL-1Ra and nearly absent by equimolar IL-1Ra.

### Activity of precursor and mature IL-33

**Figure 7:**
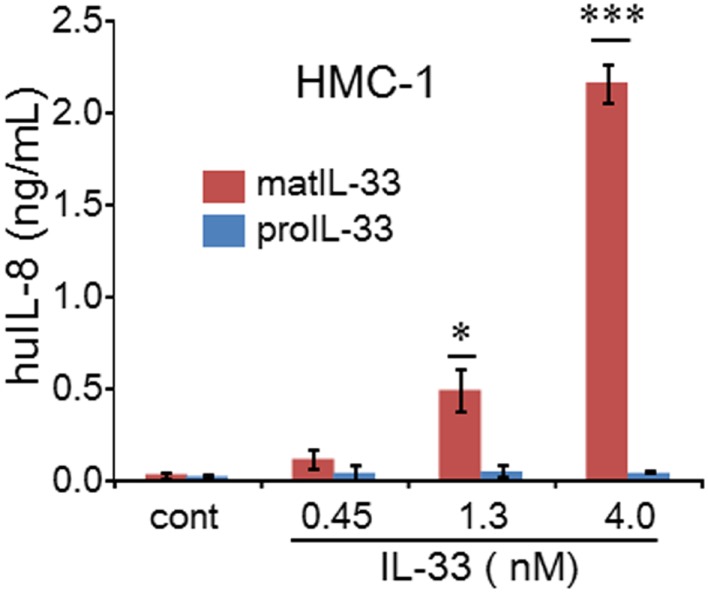
**Mean ± SEM levels of IL-8 released from human HMC-1 cells**. HMC-1 cells were stimulated for 24 h with increasing concentrations of either mature or precursor form of IL-33 as indicated under the horizontal axis (*p < 0.05; ***p < 0.001). Representative data from one of two independent experiments performed in triplicate are shown.

IL-33, another member of IL-1 family, acts as an alarmin (26–31). Precursor and mature forms of recombinant IL-33 were added to A549 cells and PBMCs. Neither precursor nor mature IL-33 were active on these cells (data not shown). In contrast, the human mast cell-1 line (HMC-1), which responds to IL-33, was assessed for activity. As shown in Figure 7, the mature form but not the precursor of IL-33 was active but did not reach statistical significance until 1.3 nM concentration.

## Discussion

Intracellular contents contain several known alarmins, each of which can initiate inflammatory responses via specific cell surface receptors; these include high mobility group box-1 (HMGB-1), members of the IL-1 family, cytosolic calcium-binding proteins of the S100 family, heat-shock proteins 70/90, S100 proteins, peroxiredoxin-1, and nucleosomes (32). For example, HMGB-1 binds to the cell surface scavenger receptor (RAGE) and IL-1α binds to the IL-1R1. Identification which and to what extent of the myriad of intracellular alarmins released from necrotic cells accounts for the inflammation is a daunting task. In the case of IL-1, specific antibody neutralization has been used to distinguish IL-1α from IL-1β (15, 16).

However, the identification does not reveal the molecular nature of the cytokine linked to biological activity. For example, when the precursors for IL-1β or IL-1α or IL-33 are released from necrotic cells into a neutrophil-rich microenvironment, extracellular processing can take place generating one or more mature forms with different levels of activity (7–9). Indeed, the biological and receptor binding activities of 23 unique amino acid sequences of mature human IL-1α (111–270) were expressed in *E. coli* and compared with WT the activity of the mature molecule. The study identified several sequences along one face of the molecule which are distinct from regions thought to bind to the IL-1 receptor (33). In the present study, we compared the biological activity of recombinant IL-1α, IL-1β, and IL-33 precursors to the activity as mature proteins using a biologically relevant assay of cytokine production from human lung epithelial A549 cells and PBMCs.

The IL-1β precursor was consistently without activity on A549 cells and PBMC whereas the mature form with the N-terminus at the caspase-1 cleavage site (34) was active. Nevertheless, *in vivo* the IL-1β precursor can be processed extracellularly by proteinase-3 and elastase into an active cytokine (9, 35). There is no caspase-1 cleavage site in the IL-1α precursor; cleavage is by a membrane associated calpain results in a 17,000 Da peptide. The N-terminus of the calpain cleavage product is assumed to be the same as that the IL-1α purified from the supernatants of a mouse cell line (36). However, it remains unclear how calpain is activated. For IL-33, extracellular processing by serine proteases generate several N-termini with varying degrees of activity (7). In the case of the precursor forms of IL-1α and IL-33, N-terminal pro-sequence has another function, binding to nucleic DNA and therefore participating in transcription (3, 5).

We also know from many studies that the IL-1α precursor is constitutive in most cells from healthy human subjects, particularly epithelial cells of the entire intestinal, respiratory, and urinary tracts as well as all keratinocytes. Thus damage or hypoxia in these tissues results in IL-1α mediated inflammation. In fact, the demonstration of the specific activity of recombinant IL-1α precursor in terms of effective concentrations supports other studies on the role of IL-1α in inducing sterile inflammation. For example, others have reported that cellular contents release by high heat (15) or prolonged exposure to hypoxic conditions (4, 16) are due to IL-1α, not IL-1β and not to TLR.

Thus, the IL-1α precursor emerges as a key alarmin from dying cells. Until the present study, there was no detailed comparison demonstrating that the IL-1α precursor can be as active as the processed form of the cytokine. This is clearly shown in Figure 2C examining the induction of IL-6 from PBMC. Others have reported that mature IL-1α is more active than the precursor using a complex assay for IL-1 that is IL-1-augementation of IL-2 production from EL4 cells (37). In that study, the investigators encountered significant difficulties with the activity of N-terminal his-tagged mature IL-1α due to aggregation. To circumvent the problem, the precursor was cleaved with calpain and the comparison of activities was made between the precursor and the calpain-generated mature IL-1α. In contract, our studies directly assessed the active recombinant forms in a direct assay on A549 cells or PBMC. In A549 cells, mature IL-1α is more active than the precursor but on PBMC, they were equally active. In terms of clinical implications of these findings, progression of inflammation with an ischemic event followed by re-perfusion, the inflammation reaches a maximal level on day 3 and at that time, IL-1β rather than IL-1α dominates the inflammation. Thus, the IL-1α precursor triggers the IL-1R1 on the resident macrophage population in the affected tissue, producing IL-1β as well as chemokines as the post-necrotic inflammatory response progresses.

In addition to sterile inflammation induced by hypoxic or ischemic events, there is role for the IL-1α precursor in model of vascular inflammation such as the vasculitis of lupus. Here the release of apoptotic bodies released dying human endothelial cells contain the precursor as well as a processed form of IL-1α (38). These apoptotic bodies are highly inflammatory *in vivo* and are due to IL-1α and not IL-1β (38). Another role for inflammation due to the IL-1α precursor is in dying tumors. As tumors outgrow their vascular supply, necrosis takes place and here the source of the IL-1α precursor is the tumor itself. Proof of this mechanism comes from a human trial in which patients with advanced cancer are treated with anti-IL-1α monoclonal human antibody. These patients are markedly cachectic and have low lean body mass. Upon treatment with anti-IL-1α, many patients start to reverse the loss in mean mass and live longer compared to untreated patients (39). The mechanism of this loss in body mass appears to be a reduction in inflammation as those patients who respond to anti-IL-1α have a fall in serum IL-6 whereas those who do not reverse the weight loss have higher IL-6 levels (39).

Not all patients responded to anti-IL-1α treatment, which raises the issue of the control of intracellular IL-1α activity in tumor cells. It is known that some forms of tissue necrosis are associated with inflammation whereas others are not (37). Thus, one can assume that the activity of IL-1α maybe under a control mechanism which is cell specific. Indeed, it has been reported that intracellular IL-1α precursor is bound to intracellular IL-1R2 and that this complex inhibits the activity of precursor IL-1α (37). In those cells without constitutive IL-1R2, the IL-1α precursor is active. One can conclude from that study that necrotic cells without constitutive IL-1R2 are likely to induce inflammation due to the activity of the IL-1α precursor whereas those with IL-1R2 are less likely to be inflammatory.

The release of alarmins such as the IL-1α precursor initially target adjacent stromal cells, resident tissue macrophages, and dendritic cells. In turn, these cells release chemokines, which then recruit the infiltration of inflammatory cells such as monocytes and neutrophils into the area of damage, thus the initial event such as ischemia in an organ increases with the infiltration of cells from the periphery. The secondary cytokines such as IL-6 released from stromal cells by the activity of the IL-1α precursor gains access to the circulation and stimulates the liver to synthesize acute phase proteins such as C reactive protein and suppress the synthesis of albumin.

Since is released from tumor cells and necrotic cells in the context of intracellular cytokines, IL-33 can be seen as a novel of alarmin (26–31). However, our data (Figure 6) show that the precursor form of IL-33 is not active on mast cells compared to the processed forms; it is likely that IL-33 has limited activities due to its receptor expression pattern in different immune cell linages including non-immune cell linage (40). Although precursor IL-33 possesses biological activity, unlike precursor IL-1α, the impact of effects could be less because only particular immune cells linages respond to IL-33. In other studies, IL-33 exhibits a weak activity as a precursor compared to mature IL-33 (6, 19, 40).

In summary, our data of precursor and mature recombinant IL-1α, IL-1β, and IL-33 proteins demonstrate that the precursor from of IL-1α is a key alarmin since precursor protein without the processing of maturation such as inflammasome dependent or independent pathways. The biological activity of IL-1α as an alarmin is due to the rapid release of intracellular molecules in the absence of processing mechanisms, this initiating downstream inflammation. Unlike precursor IL-1α, precursor IL-1β, and IL-33 failed to exhibit biological activities though mature IL-1β and IL-33 were highly active.

## Conflict of Interest Statement

The authors declare that the research was conducted in the absence of any commercial or financial relationships that could be construed as a potential conflict of interest.
